# Is Higher Viral Load in the Upper Respiratory Tract Associated With Severe Pneumonia? Findings From the PERCH Study

**DOI:** 10.1093/cid/cix148

**Published:** 2017-05-29

**Authors:** Daniel R. Feikin, Wei Fu, Daniel E. Park, Qiyuan Shi, Melissa M. Higdon, Henry C. Baggett, W. Abdullah Brooks, Maria Deloria Knoll, Laura L. Hammitt, Stephen R. C. Howie, Karen L. Kotloff, Orin S. Levine, Shabir A. Madhi, J. Anthony G. Scott, Donald M. Thea, Peter V. Adrian, Martin Antonio, Juliet O. Awori, Vicky L. Baillie, Andrea N. DeLuca, Amanda J. Driscoll, Bernard E. Ebruke, Doli Goswami, Ruth A. Karron, Mengying Li, Susan C. Morpeth, John Mwaba, James Mwansa, Christine Prosperi, Pongpun Sawatwong, Samba O. Sow, Milagritos D. Tapia, Toni Whistler, Khalequ Zaman, Scott L. Zeger, Katherine L. O’ Brien, David R. Murdoch, for the PERCH Study Group, Katherine L. O’Brien, Orin S. Levine, Maria Deloria Knoll, Daniel R. Feikin, Andrea N. DeLuca, Amanda J. Driscoll, Nicholas Fancourt, Wei Fu, Laura L. Hammitt, Melissa M. Higdon, E. Wangeci Kagucia, Ruth A. Karron, Mengying Li, Daniel E. Park, Christine Prosperi, Zhenke Wu, Scott L. Zeger, Nora L. Watson, Jane Crawley, David R. Murdoch, W. Abdullah Brooks, Hubert P. Endtz, Khalequ Zaman, Doli Goswami, Lokman Hossain, Yasmin Jahan, Hasan Ashraf, Stephen R. C. Howie, Bernard E. Ebruke, Martin Antonio, Jessica McLellan, Eunice Machuka, Arifin Shamsul, Syed M.A. Zaman, Grant Mackenzie, J. Anthony G. Scott, Juliet O. Awori, Susan C. Morpeth, Alice Kamau, Sidi Kazungu, Micah Silaba Ominde, Karen L. Kotloff, Milagritos D. Tapia, Samba O. Sow, Mamadou Sylla, Boubou Tamboura, Uma Onwuchekwa, Nana Kourouma, Aliou Toure, Shabir A. Madhi, David P. Moore, Peter V. Adrian, Vicky L. Baillie, Locadiah Kuwanda, Azwifarwi Mudau, Michelle J. Groome, Nasreen Mahomed, Henry C. Baggett, Somsak Thamthitiwat, Susan A. Maloney, Charatdao Bunthi, Julia Rhodes, Pongpun Sawatwong, Pasakorn Akarasewi, Donald M. Thea, Lawrence Mwananyanda, James Chipeta, Phil Seidenberg, James Mwansa, Somwe wa Somwe, Geoffrey Kwenda, Trevor P. Anderson, Joanne Mitchell

**Affiliations:** 1 Department of International Health, International Vaccine Access Center, Johns Hopkins Bloomberg School of Public Health, Baltimore, Maryland; 2 Division of Viral Diseases, National Center for Immunization and Respiratory Diseases, Centers for Disease Control and Prevention, Atlanta, Georgia; 3 Department of Rheumatology, Johns Hopkins School of Medicine, Baltimore, Maryland;; 4 Milken Institute School of Public Health, Department of Epidemiology and Biostatistics, George Washington University, District of Columbia;; 5 Global Disease Detection Center, Thailand Ministry of Public Health–US Centers for Disease Control and Prevention Collaboration, Nonthaburi;; 6 Division of Global Health Protection, Center for Global Health, Centers for Disease Control and Prevention, Atlanta, Georgia;; 7 International Centre for Diarrhoeal Disease Research, Bangladesh (icddr,b), Dhaka and Matlab;; 8 Department of International Health, Johns Hopkins Bloomberg School of Public Health, Baltimore, Maryland;; 9 Kenya Medical Research Institute–Wellcome Trust Research Programme, Kilifi;; 10 Medical Research Council Unit, Basse, The Gambia;; 11 Department of Paediatrics, University of Auckland, and; 12 Centre for International Health, University of Otago, Dunedin, New Zealand;; 13 Division of Infectious Disease and Tropical Pediatrics, Department of Pediatrics, Center for Vaccine Development, Institute of Global Health, University of Maryland School of Medicine, Baltimore;; 14 Bill & Melinda Gates Foundation, Seattle, Washington;; 15 Medical Research Council, Respiratory and Meningeal Pathogens Research Unit, and; 16 Department of Science and Technology/National Research Foundation, Vaccine Preventable Diseases Unit, University of the Witwatersrand, Johannesburg, South Africa;; 17 Department of Infectious Disease Epidemiology, London School of Hygiene & Tropical Medicine, United Kingdom;; 18 Center for Global Health and Development, Boston University School of Public Health, Massachusetts;; 19 Department of Pathogen Molecular Biology, London School of Hygiene & Tropical Medicine, and; 20 Microbiology and Infection Unit, Warwick Medical School, University of Warwick, Coventry, United Kingdom;; 21 Department of Epidemiology,; 22 Department of International Health, Center for Immunization Research, and; 23 Department of Population, Family and Reproductive Health, Johns Hopkins Bloomberg School of Public Health, Baltimore, Maryland;; 24 Microbiology Laboratory, Middlemore Hospital, Counties Manukau District Health Board, Auckland, New Zealand;; 25 Department of Pathology and Microbiology, University Teaching Hospital, and; 26 Zambia Center for Applied Health Research and Development, Lusaka;; 27 Centre pour le Développement des Vaccins (CVD-Mali), Bamako;; 28 Department of Biostatistics, Johns Hopkins Bloomberg School of Public Health, Baltimore, Maryland;; 29 Department of Pathology, University of Otago, and; 30 Microbiology Unit, Canterbury Health Laboratories, Christchurch, New Zealand; 31 Johns Hopkins Bloomberg School of Public Health, Baltimore, Maryland; 32 Bill & Melinda Gates Foundation, Seattle, Washington; 33 Centers for Disease Control and Prevention [CDC], Atlanta, Georgia; 34 Emmes Corporation, Rockville, Maryland; 35 Nuffield Department of Clinical Medicine, University of Oxford, United Kingdom; 36 University of Otago, Christchurch, New Zealand; 37 icddr,b, Dhaka and Matlab, Bangladesh; 38 Medical Research Council, Basse, The Gambia; 39 KEMRI–Wellcome Trust Research Programme, Kilifi, Kenya; 40 Division of Infectious Disease and Tropical Pediatrics, Department of Pediatrics, Center for Vaccine Development, Institute of Global Health, University of Maryland School of Medicine, Baltimore, Maryland and Centre pour le Développement des Vaccins (CVD-Mali), Bamako, Mali; 41 Respiratory and Meningeal Pathogens Research Unit, University of the Witwatersrand, Johannesburg, South Africa; 42 Thailand Ministry of Public Health–US CDC Collaboration, Nonthaburi, Thailand; 43 Boston University School of Public Health, Boston, Massachusetts and University Teaching Hospital, Lusaka, Zambia; 44 Canterbury Health Laboratory, Christchurch, New Zealand

**Keywords:** pneumonia, viral load, viral density, RSV, PERCH.

## Abstract

**Background.:**

The etiologic inference of identifying a pathogen in the upper respiratory tract (URT) of children with pneumonia is unclear. To determine if viral load could provide evidence of causality of pneumonia, we compared viral load in the URT of children with World Health Organization–defined severe and very severe pneumonia and age-matched community controls.

**Methods.:**

In the 9 developing country sites, nasopharyngeal/oropharyngeal swabs from children with and without pneumonia were tested using quantitative real-time polymerase chain reaction for 17 viruses. The association of viral load with case status was evaluated using logistic regression. Receiver operating characteristic (ROC) curves were constructed to determine optimal discriminatory viral load cutoffs. Viral load density distributions were plotted.

**Results.:**

The mean viral load was higher in cases than controls for 7 viruses. However, there was substantial overlap in viral load distribution of cases and controls for all viruses. ROC curves to determine the optimal viral load cutoff produced an area under the curve of <0.80 for all viruses, suggesting poor to fair discrimination between cases and controls. Fatal and very severe pneumonia cases did not have higher viral load than less severe cases for most viruses.

**Conclusions.:**

Although we found higher viral loads among pneumonia cases than controls for some viruses, the utility in using viral load of URT specimens to define viral pneumonia was equivocal. Our analysis was limited by lack of a gold standard for viral pneumonia.

For diagnosing viral pneumonia, upper respiratory tract (URT) specimens have become the most common specimen type due to their logistical ease of collection [1, 2]. However, detection of viruses in URT specimens has low specificity as this finding might simply reflect an URT infection without lower respiratory tract involvement or coincidental asymptomatic or past infection [2–4].

A possible solution to the lack of specificity of simply detecting the presence or absence of a virus in the URT of pneumonia patients is to determine whether the density of a virus in the URT can better distinguish its causative role in pneumonia. There are reports that a higher pathogen load in the URT is associated with pneumonia, for both *Streptococcus pneumoniae* and some respiratory viruses [5–8]. In addition, for some viruses, higher viral load in the URT has been associated with worse outcomes [7, 9–11].

In this analysis, we describe viral load in nasopharyngeal/oropharyngeal (NP/OP) specimens from cases and community controls from a large multicountry childhood pneumonia study (Pneumonia Etiology Research for Child Health [PERCH]), as well as demographic and clinical characteristics associated with higher viral load and disease severity. An overarching aim was to explore whether the incorporation of viral load data into the main PERCH etiology analysis might improve the assignment of the etiology of pneumonia cases.

## METHODS

The PERCH study design and enrollment strategy has been previously described [12]. In brief, PERCH is a case-control study of the etiology of World Health Organization (WHO)–defined severe and very severe pneumonia among hospitalized children aged 1–59 months and age frequency-matched community controls. Enrollment took place during August 2011–January 2014 for 24 months at each of 9 study sites located in 7 countries—Dhaka and Matlab, Bangladesh; Basse, The Gambia; Kilifi, Kenya; Bamako, Mali; Soweto, South Africa; Nakhon Phanom and Sa Kaeo, Thailand; and Lusaka, Zambia [13].

### Case and Control Definitions

For this analysis, we included only cases with evidence of pneumonia on chest radiograph, defined as consolidation and/or any other infiltrate assessed according to the WHO radiological pneumonia criteria [14]. A control participant was considered to have a respiratory tract illness (RTI) if cough or runny nose was reported. RTI was also considered present if a child had (1) at least 1 of ear discharge, wheezing, or difficulty breathing and (2) either a measured temperature of ≥38.0°C within the previous 48 hours or a history of sore throat. Controls who did not meet the definition of RTI are referred to as non-RTI controls.

### Specimen Collection and Laboratory Testing

Nasopharyngeal and oropharyngeal swabs were collected from PERCH cases and controls at enrollment. Nasopharyngeal specimens were collected by inserting flocked swabs (Copan ETC) into the posterior nasopharynx and rotating 180° for 2–3 seconds. Oropharyngeal specimens were then collected by rubbing Rayon swabs (Fisher Scientific) over both tonsillar pillars and the posterior oropharynx for several seconds. Following collection, swabs were placed together in the same 3-mL vial of universal transport media (Copan) and processed within 24 hours of collection. Specimens were left at room temperature for no more than 2 hours or at 4°C for no more than 24 hours, before freezing at –70°C.

All specimens were tested in-country using a standardized methodology, and details are described elsewhere [15]. Specimens were evaluated using the Fast-track Diagnostics Respiratory Pathogens 33 test (FTD Resp 33, Fast-track Diagnostics, Sliema, Malta), a 33-target, 8-multiplex real-time polymerase chain reaction (PCR) platform for the detection of viruses, bacteria, and fungi. The 18 viruses or virus classes included influenza A, B, and C viruses; parainfluenza virus (PIV) types 1, 2, 3, and 4; coronaviruses NL63, 229E, OC43, and HKU1; human metapneumovirus (HMPV) A and B (A and B not differentiated); rhinovirus; respiratory syncytial virus (RSV) A and B (A and B not differentiated); adenovirus; enterovirus and parechovirus (not differentiated); human bocavirus (HBOV); and cytomegalovirus. Cytomegalovirus is not included in this analysis but is discussed in a separate publication of the pathogen load of pathogens commonly detected in both cases and controls [16].

Some sites (Bangladesh, The Gambia, Mali, South Africa) collected lung aspirates from children with consolidation on chest radiograph who met clinical and radiologic criteria for the procedure [17]. Lung aspirate specimens were tested for viral targets using the same method described for NP/OP specimens.

### Statistical Analysis

Human immunodeficiency virus (HIV)–positive cases were excluded from analyses unless stated otherwise. PCR quantification was log_10_-transformed. Demographic characteristics of cases and controls were compared using the χ^2^ test. All controls, both RTI and non-RTI, were included in the main analysis. All analyses of viral load were restricted to children positive for each virus. Among children positive for each virus, *t* tests adjusted for site and age were performed to compare mean cycle threshold (Ct) values between cases and controls. For each virus, a trend analysis, using simple linear regression, was performed to test if viral density increased with age for cases and for controls. Among cases only, mean Ct values were also compared by days since onset, severity, vital status, and HIV status. Multivariable logistic regression, adjusting for age and site, was performed to compare odds of being a case for each 3.4-unit drop in Ct value, which was approximately equivalent to a 1 log_10_ increase in viral copies/mL; Ct values instead of viral density were used for regression because viral density was only accurate within the linear range of the assay (10^4^–10^8^ copies/mL). Kernel density distribution plots were created to show distributions of viral density by case/control status. Receiver operating characteristic (ROC) curves and the corresponding area under the curve (AUC) were generated to investigate the performance of viral load in determining case status among children positive by NP/OP PCR for each virus, and the Youden index was calculated to determine the optimized diagnostic cutoffs to differentiate cases and controls [18]. To guard against bias in the estimates of sensitivity due to having a small number of some viruses detected among cases, the Youden index was calculated using leave-one-out cross-validation where sample size was sufficient [19]. Redefining positivity using the optimal cutpoints, we calculated odds ratios associated with case status for children above vs below the optimal cutpoint including negatives. The proportion of radiographically confirmed cases attributable to each virus [population attributable fraction: population prevalence × (1 – 1 / OR)] was calculated using 2 methods: (1) any positive vs negative and (2) positive above vs below the optimal cutpoint, the former method being optimal for laboratory sensitivity and the latter for a balance of epidemiological sensitivity and specificity. All analyses were performed using SAS software version 9.4 (SAS Institute, Cary North Carolina) and R Statistical Software 3.2.1 (R Foundation for Statistical Computing, Vienna, Austria). All *P* values are 2 sided.

### Ethical Considerations

The PERCH study protocol was approved by the institutional review board or ethical review committee at each of the study site institutions and at the Johns Hopkins Bloomberg School of Public Health. Parents or guardians of all participants provided written informed consent.

## RESULTS

Of 1935 radiographically confirmed cases and 5325 controls in PERCH, 1733 cases (1227 severe, 506 very severe) and 4986 controls (1185 RTI and 3801 non-RTI) were not known to be HIV infected and had viral density results available for analysis (Table 1).

**Table 1.** Characteristics of Chest Radiograph–Positive Children With Severe and Very Severe Pneumonia and Controls—Pneumonia Etiology Research for Child Health (PERCH) Study, August 2011–January 2014

Characteristic	CXR+Cases^a^ (n = 1733)	All Controls (n = 4986)	χ^2^*P* Value^b^
Site			
Kenya	282 (16.3)	855 (17.2)	**<.001**
The Gambia	273 (15.8)	624 (12.5)	
Mali	239 (13.8)	724 (14.5)	
Zambia	189 (10.9)	535 (10.7)	
South Africa	433 (25.0)	823 (16.5)	
Thailand	98 (5.7)	657 (13.2)	
Bangladesh	219 (12.6)	768 (15.4)	
Age			
1–5 mo	680 (39.2)	1555 (31.2)	**<.001**
6–11 mo	415 (24.0)	1187 (23.8)	
12–23 mo	424 (24.5)	1235 (24.8)	
24–59 mo	214 (12.4)	1009 (20.2)	
Female sex	756 (43.6)	2477 (49.7)	**<.001**
Prior antibiotic use^c^	597 (42.4)	84 (1.7)	**<.001**
Respiratory tract illness^d^	NA	1185 (23.8)	NA
No. of viruses detected			
0 viruses	180 (10.4)	1048 (21.0)	
1 virus	628 (36.2)	1928 (38.7)	**<.001**
2 viruses	616 (35.6)	1420 (28.5)	
≥3 viruses	309 (17.8)	590 (11.8)	

Data are presented as No. (%) unless otherwise indicated.

Abbreviations: CXR^+^, chest radiograph positive; NA, not applicable.

^a^CXR^+^ defined as having radiographic evidence of pneumonia.

^b^Comparing distribution of characteristics between CXR^+^ cases and controls. Bolded values are significant (*P* < .05).

^c^Prior antibiotic use: administered antibiotics at the study facility prior to the collection of specimens (cases only), antibiotics at a referral facility (cases only), or positive serum bioassay (cases and controls).

^d^See Methods for respiratory tract illness definition.

Overall, 89.6% of cases and 79.0% of controls had at least 1 virus detected, and 53.4% and 40.3%, respectively, had ≥2 viruses detected (Table 1). Among the 17 viruses tested, RSV was the most commonly detected among cases (27%) but was uncommon among controls (3%) (Table 2). Rhinovirus was the next most commonly detected virus in cases but was present at a similar frequency among controls (21% for both).

**Table 2.** Mean Nasopharyngeal/Oropharyngeal Polymerase Chain Reaction Cycle Threshold Values for Chest Radiograph–Positive Cases and Controls and Odds Ratios for Viral Load Being Predictive of Case Status—Pneumonia Etiology Research for Child Health (PERCH) Study, August 2011–January 2014

Virus	CXR+ Cases^a^ (n = 1733)			All Controls (n = 4986)			*P* Value^d^	OR per 1 Log_10_ Increase, Copies/mL (95% CI)^e^
	No.^b^	(%)^b^	Ct Value Mean^c^ (95% CI)	No.^b^	(%)^b^	Ct Value Mean^c^ (95% CI)		
Adenovirus	164	(9.5)	27.7 (26.8–28.5)	594	(11.9)	29.5 (29.2–29.8)	**<.001**	**1.27 (1.10–1.46**)
Coronavirus 229	18	(1.1)	31.1 (28.6–33.5)	54	(1.1)	30.2 (28.3–32.0)	.58	0.89 (.62–1.26)
Coronavirus 43	38	(2.2)	26.4 (24.4–28.5)	192	(3.9)	28.0 (27.1–28.8)	.30	1.13 (.91–1.39)
Coronavirus 63	36	(2.1)	27.0 (25.3–28.7)	158	(3.2)	28.5 (27.7–29.3)	.26	1.18 (.90–1.55)
Coronavirus HKU	37	(2.2)	29.2 (27.0–31.4)	111	(2.2)	27.7 (26.5–28.9)	.40	0.91 (.74–1.13)
Influenza A	62	(3.6)	28.5 (27.7–29.4)	57	(1.2)	29.8 (28.4–31.2)	.31	1.21 (.85–1.72)
Influenza B	18	(1.1)	27.6 (25.7–29.5)	29	(0.6)	28.5 (26.7–30.3)	.82	1.07 (.63–1.83)
Influenza C	10	(0.6)	28.1 (24.8–31.4)	29	(0.6)	27.3 (25.3–29.3)	.14	0.44 (.17–1.15)
HBOV	231	(13.4)	30.5 (29.6–31.3)	660	(13.3)	31.7 (31.3–32.1)	**.007**	**1.13 (1.03–1.24**)
HMPV A/B	185	(10.8)	28.1 (27.6–28.7)	206	(4.1)	28.9 (28.2–29.5)	**.02**	**1.23 (1.03–1.46**)
Parainfluenza 1	89	(5.2)	26.1 (24.9–27.2)	49	(1.0)	29.4 (27.6–31.2)	**.008**	**1.37 (1.08–1.74**)
Parainfluenza 2	23	(1.3)	34.0 (31.7–36.3)	53	(1.1)	35.1 (33.9–36.3)	.70	1.10 (.71–1.69)
Parainfluenza 3	104	(6.1)	25.0 (24.0–25.9)	142	(2.9)	29.0 (28.0–30.0)	**<.001**	**1.47 (1.22–1.77**)
Parainfluenza 4	44	(2.6)	31.7 (30.3–33.1)	86	(1.7)	32.2 (31.3–33.1)	.88	0.98 (.73–1.31)
PV/EV	131	(7.6)	30.1 (29.5–30.8)	423	(8.5)	30.4 (30.0–30.7)	.45	1.08 (.89–1.31)
Rhinovirus	365	(21.2)	31.7 (31.3–32.0)	1056	(21.2)	32.4 (32.3–32.6)	**.003**	**1.21 (1.08–1.35**)
RSV	461	(26.8)	22.2 (21.8–22.5)	140	(2.8)	27.0 (26.1–28.0)	**<.001**	**2.02 (1.71–2.37**)

Abbreviations: CI, confidence interval; Ct, cycle threshold; CXR+, chest radiograph positive; HBOV, human bocavirus; HMPV, human metapneumovirus; OR, odds ratio; PV/EV, parechovirus/enterovirus; RSV, respiratory syncytial virus.

^a^CXR+ defined as having radiographic evidence of pneumonia.

^b^No. (%) positive in the nasopharynx/oropharynx among those with available results for the given virus.

^c^Among those with a positive density.

^d^Comparing mean cycle threshold value of CXR+ cases vs all controls using linear regression adjusting for age and site. Bolded values are significant (*P* < .05).

^e^Odds ratio is for approximately each 3.4-unit drop in Ct value (equivalent to approximately 1 log_10_ increase in copies/mL) adjusting for age and site using logistic regression. Bolded values are significant (*P* < .05).

### Analysis of Viral Load Among Cases and Controls

**Figure 1.**
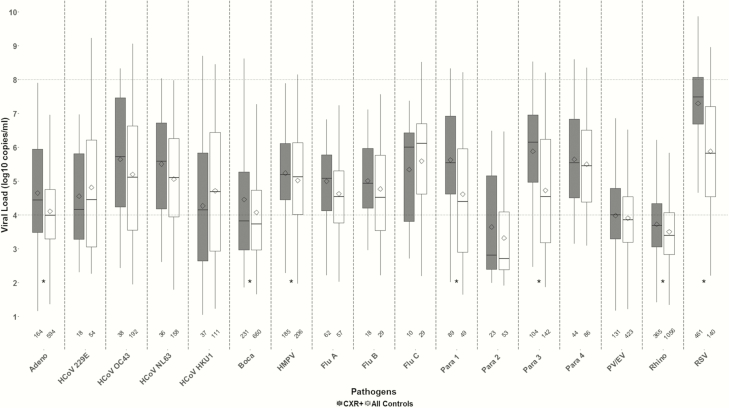
Nasopharyngeal/oropharyngeal viral load (log_10_ copies/mL) for chest radiograph–positive (CXR+) cases and all controls among those in which the virus was detected—Pneumonia Etiology Research for Child Health (PERCH) study, August 2011–January 2014. CXR+ defined as having radiographic evidence of pneumonia. Box-and-whiskers plot features include the following: central line in box is median, bottom line of box is first quartile (25%), top line of box is third quartile (75%), diamond is mean, and top and bottom of whiskers represent 95% confidence intervals. Area above the upper dotted line and below the lower dotted line indicate areas outside the linear range of the assay for calculation of viral load from cycle threshold (Ct) values where there is a greater degree of uncertainty in viral density calculations. Numbers on x-axis indicate number of positive results for that virus. *P value comparing mean Ct value between controls and CXR+ cases <.05 after adjusting for age and site. Abbreviations: Adeno, adenovirus; Boca, human bocavirus; CXR, chest radiograph; Flu, influenza virus; HCoV, human coronavirus; HMPV, human metapneumovirus; Para, parainfluenza virus; PV/EV, parechovirus/enterovirus; Rhino, rhinovirus; RSV, respiratory syncytial virus.

RSV had the highest mean viral load among cases (7.3 log copies/mL; Figure 1); no viruses other than RSV had a mean viral load >6 log copies/mL. Among controls, no viruses had a mean viral load >6 log copies/mL. Eight viruses among cases (RSV, influenza C, PIV1, PIV3, PIV4, coronavirus 43, coronavirus 63, and HMPV) had mean viral loads >5 log copies/mL vs 5 viruses among controls (RSV, influenza C, PIV4, coronavirus 43, and coronavirus 63). There were 7 viruses that had significantly higher mean viral density among cases after adjusting for site and age—adenovirus, HBOV, HMPV, PIV1, PIV3, rhinovirus, and RSV. After adjusting for age and site, there was a significant increase in the odds of being a case (vs a control) for each 3.4-unit drop in Ct value (approximately 1 log increase in copies/mL) for the same 7 viruses, ranging from a 13% increased odds for HBOV to a 102% increased odds for RSV (Table 2).

Viral load was similar between RTI and non-RTI controls for most viruses with the exception of RSV, where the mean viral load was significantly higher for RTI controls (Supplementary Figure 1).

**Figure 2.**
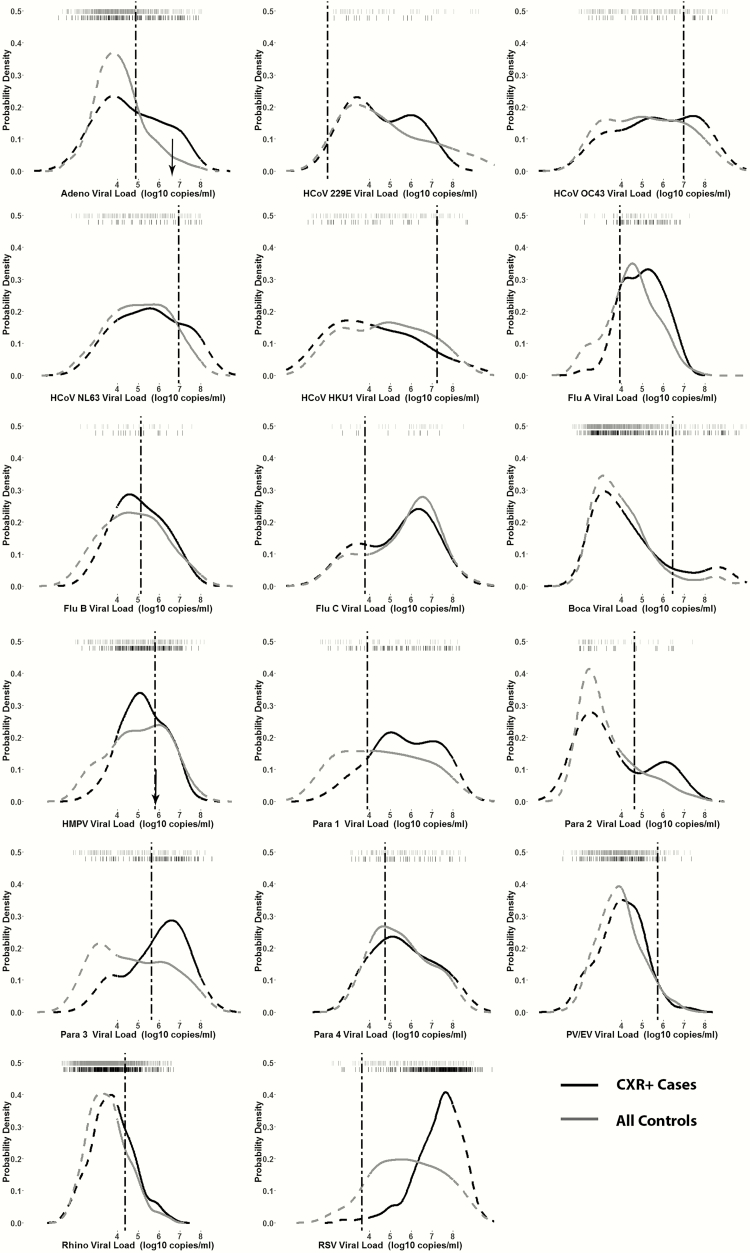
Kernel density distribution plots comparing nasopharyngeal/oropharyngeal (NP/OP) viral load among chest radiograph–positive (CXR+) cases and all controls for each viral polymerase chain reaction (PCR) target—Pneumonia Etiology Research for Child Health (PERCH) study, August 2011–January 2014. Tick marks across the top of each plot indicate viral load of each individual (first row of black ticks for cases and second row of gray ticks for controls). Dashed curves indicate areas outside the linear range of the assay for calculation of viral load from cycle threshold values. Dotted dashed vertical lines indicate optimal cutpoint distinguishing cases and controls calculated using Youden index. Black arrows in adenovirus and human metapneumovirus plots indicate NP/OP viral load of cases whose lung aspirate specimen was available and PCR positive for that virus. Abbreviations: Adeno, adenovirus; Boca, human bocavirus; CXR, chest radiograph; Flu, influenza virus; HCoV, human coronavirus; HMPV, human metapneumovirus; Para, parainfluenza virus; PV/EV, parechovirus/enterovirus; Rhino, rhinovirus; RSV, respiratory syncytial virus.

Despite the differences in viral load between cases and controls noted above, there was substantial overlap in the viral density distribution between cases and controls in which virus was detected, as shown in the box plots and kernel density distribution plots (Figures 1 and 2). Kernel density distribution plots were examined for a bimodal distribution with a smaller subset of cases having viruses at a higher viral load that might be indicative of those with pneumonia due to that virus. Adenovirus, coronavirus 229, and PIV1–3 had a suggestion of a bimodal distribution among cases. The NP/OP viral load for the 2 cases with viruses detected in PCR of lung aspirates (ie, HMPV and adenovirus) fell within the distribution of viral loads for other cases, as well as controls, positive for that virus (Figure 2).

**Figure 3.**
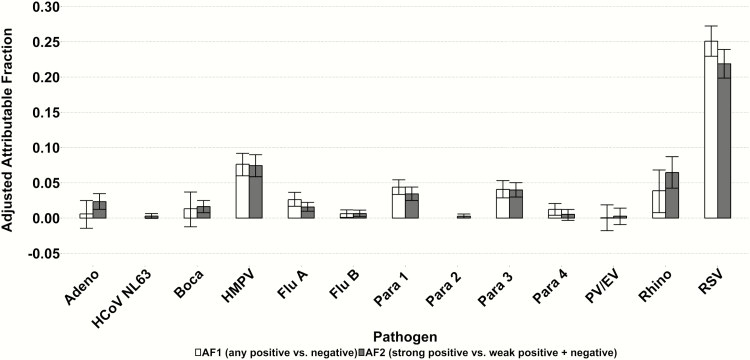
Adjusted population attributable fraction (PAF) for chest radiograph–positive cases using 2 methods: any positive vs negative (AF1) and positive above optimal cutpoint vs positive below optimal cutpoint and negative (AF2)— Pneumonia Etiology Research for Child Health (PERCH) study, August 2011–January 2014. PAF **=** population prevalence × (1 – 1 / odds ratio). Odds ratio (OR) is adjusted for other viruses, site, and age. Confidence intervals calculated using bootstrapping method. PAF not shown where adjusted OR was <1 resulting in negative PAF. Abbreviations: Adeno, adenovirus; AF, attributable fraction; Boca, human bocavirus; Flu, influenza virus; HCoV, human coronavirus; HMPV, human metapneumovirus; Para, parainfluenza virus; PV/EV, parechovirus/enterovirus.

When constructing ROC curves, no virus had an AUC >0.8 (Table 3). RSV had the highest AUC at 0.76, with only 3 other viruses having an AUC between 0.6 and 0.7 (influenza A, PIV1, and PIV3). Despite the low values for the AUC, when redefining positive for a virus as those with viral loads above the ROC optimal cutpoint value as determined by the Youden index, the odds ratio for predicting case status increased substantially for some viruses, approximately doubling for adenovirus, coronavirus 63, PIV2, and RSV (Table 4). Although the odds ratios increased, the population attributable fraction for most viruses did not change substantially, or even decreased (eg, influenza A, RSV) due to the lower frequency of cases with densities above the optimal cutpoint (Figure 3). This is because while the odds ratios are higher at the higher density cutoff, the prevalence of cases above the higher threshold was lower, and thus the proportion of PERCH cases assigned to the virus would not change appreciably by using the higher cutoff.

**Table 3.** Receiver Operating Characteristic Areas Under the Curve, Optimal Nasopharyngeal/Oropharyngeal Polymerase Chain Reaction Density Cutpoints for Determining Case Status, and Associated Positive Rate in Cases and Negative Rate in Controls by Virus Among Chest Radiograph–Positive Cases and Controls With Positive Densities—Pneumonia Etiology Research for Child Health (PERCH) Study, August 2011–January 2014

Virus^a^	AUC	Optimal Cutpoint^b^, (Log_10_ Copies/ mL)	Proportion of CXR+^c^ Cases Above Cutpoint	Proportion of Controls Below Cutpoint
Adenovirus	0.60	4.88	0.44	0.78
Coronavirus 43	0.57	6.94	0.36	0.74
Coronavirus 63	0.58	7.24	0.22	0.89
Influenza A	0.61	5.12	0.50	0.68
Influenza B	0.55	3.79	0.89	0.28
HBOV	0.54	5.81	0.20	0.89
HMPV A/B	0.54	3.9	0.91	0.20
Parainfluenza 1	0.65	4.62	0.75	0.55
Parainfluenza 2	0.54	5.64	0.26	0.91
Parainfluenza 3	0.69	4.75	0.81	0.54
PV/EV	0.53	4.38	0.40	0.71
Rhinovirus	0.56	3.64	0.55	0.56
RSV	0.76	6.30	0.84	0.59

Abbreviations: AUC, area under the curve; CXR+, chest radiograph positive; HBOV, human bocavirus; HMPV, human metapneumovirus; PV/EV, parechovirus/enterovirus; RSV, respiratory syncytial virus.

^a^Viruses with adjusted odds ratios <1 (see Table 2) were excluded from table (coronavirus 229, coronavirus HKU, influenza C, and parainfluenza 4).

^b^Calculated using Youden index and, where possible, leave-one-out cross-validation. Leave-one-out cross-validation was not performed for influenza B, parainfluenza 2, or parechovirus/enterovirus.

^c^CXR+ defined as having radiographic evidence of pneumonia.

**Table 4.** Nasopharyngeal/Oropharyngeal Prevalence of Viruses in Chest Radiograph–Positive Cases and Controls, Defining Positive as Any Detection of Virus and Detection of Virus Above an Optimal Viral Load Cutpoint as Determined by Receiver Operating Characteristic Curves; Odds of Determining Case Status Using Both Definitions of Positive— Pneumonia Etiology Research for Child Health (PERCH) Study, August 2011–January 2014

Virus^a^	Negative		Weak Positive^c^ (Below Optimal Cutpoint)		Strong Positive^d^ (Above Optimal Cutpoint)		AOR^e^ (95% CI) Any Positive vs Negative^f^	AOR^e^ (95% CI) Strong Positive vs Weak Positive/ Negative^g^
	CXR+ Cases^b^	Controls	CXR+ Cases^b^	Controls	CXR+ Cases^b^	Controls		
Adenovirus	1556 (90.5)	4384 (88.0)	92 (5.3)	470 (9.4)	72 (4.2)	126 (2.5)	0.88 (.73–1.07)	**1.74 (1.28**–**2.36**)
Coronavirus 43	1681 (97.7)	4785 (96.1)	25 (1.5)	149 (3.0)	14 (0.8)	43 (0.9)	**0.52 (.36**–**.74**)	0.81 (.44–1.49)
Coronavirus 63	1684 (97.9)	4819 (96.8)	28 (1.6)	147 (3.0)	8 (0.5)	11 (0.2)	**0.63 (.43**–**.91**)	1.78 (.71–4.47)
Influenza A	1658 (96.4)	4920 (98.9)	31 (1.8)	41 (0.8)	31 (1.8)	16 (0.3)	**3.11 (2.15**–**4.49**)	**5.32 (2.87**–**9.85**)
Influenza B	1702 (99.0)	4948 (99.4)	2 (0.1)	8 (0.2)	16 (0.9)	21 (0.4)	1.82 (1.00–3.32)	**2.24 (1.15**–**4.36**)
HBOV	1488 (86.6)	4316 (86.7)	184 (10.7)	593 (11.9)	47 (2.7)	68 (1.4)	1.11 (.94–1.31)	**2.02 (1.38**–**2.96**)
HMPV A/B	1534 (89.2)	4771 (95.9)	16 (0.9)	47 (0.9)	169 (9.8)	159 (3.2)	**2.59 (2.09**–**3.21**)	**3.02 (2.40**–**3.82**)
Parainfluenza 1	1630 (94.8)	4928 (99.0)	23 (1.3)	27 (0.5)	66 (3.8)	22 (0.4)	**5.19 (3.60**–**7.49**)	**8.09 (4.92**–**13.32**)
Parainfluenza 2	1697 (98.7)	4927 (98.9)	18 (1.0)	48 (1.0)	5 (0.3)	5 (0.1)	1.2 (.73–1.97)	2.55 (.72–9.04)
Parainfluenza 3	1616 (94.0)	4838 (97.1)	21 (1.2)	78 (1.6)	83 (4.8)	64 (1.3)	**2.13 (1.63**–**2.77**)	**3.52 (2.51**–**4.92**)
PV/EV	1589 (92.4)	4555 (91.5)	79 (4.6)	303 (6.1)	52 (3.0)	122 (2.4)	0.91 (.74–1.12)	1.22 (.87–1.71)
Rhinovirus	1260 (73.3)	3675 (73.8)	211 (12.3)	743 (14.9)	249 (14.5)	559 (11.2)	0.94 (.82–1.07)	**1.26 (1.07**–**1.48**)
RSV	1259 (73.2)	4840 (97.2)	73 (4.2)	83 (1.7)	388 (22.6)	57 (1.1)	**12.55 (10.24**–**15.38**)	**24.72 (18.52**–**33.01**)

Data are presented as No. (%) unless otherwise indicated.

Abbreviations: AOR, adjusted odds ratio; CI, confidence interval; CXR+, chest radiograph positive; HBOV, human bocavirus; HMPV, human metapneumovirus; PV/EV, parechovirus/enterovirus; RSV, respiratory syncytial virus.

^a^Viruses with adjusted odds ratios <1 for association of density (log copies/mL) with case status excluded from table (coronavirus 229, coronavirus HKU, influenza C, and parainfluenza virus 4 as noted in Table 2).

^b^CXR+ defined as having radiographic evidence of pneumonia.

^c^Weakly positive: positive density below optimal cutpoint determined by Youden index. See Table 3.

^d^Strongly positive: density above optimal cutpoint determined by Youden index. See Table 3.

^e^Odds ratios adjusted for site and age. Bolded values are significant (*P* < .05).

^f^Any positive includes those below and above optimal cutoff.

^g^Strong positives are compared with combined negatives and weak positives.

### Predictors of Viral Load

We explored several potential predictors of viral density among cases. When NP/OP specimens were collected earlier in the course of illness, mean viral load was higher for RSV, PIV1, and PIV3 and was lower for adenovirus; no significant difference was observed for the other viruses (Supplementary Table 1). The viral load among cases did not vary by age for most viruses, including RSV, but a significant trend toward decreasing viral load with increasing age was observed for a few viruses, including adenovirus and RSV, which was also observed among controls (Supplementary Figure 2). A slight, but significant trend toward increasing viral load with increasing age was seen for rhinovirus among cases, but a significant trend in the opposite direction was observed for controls. In general, viral load did not vary by study site. One notable exception was higher PIV1 viral load in The Gambia site (the only site with a PIV1 outbreak, data not shown). HIV-infected cases had a higher mean viral load for coronavirus 43 and a lower viral load for HMPV, PIV3, and RSV (Supplementary Table 2). There were no significant differences in the viral load between cases who were normally nourished vs malnourished (except PIV1 viral load was higher in normally nourished, data not shown).

We assessed whether viral load was associated with pneumonia severity. Rhinovirus was the only virus with higher mean viral load for very severe pneumonia compared to severe pneumonia (Supplementary Figure 3). Influenza A was the only virus with higher mean viral load in fatal compared with surviving cases (Supplementary Figure 4). Furthermore, we compared mean viral densities between cases with evidence of an other infiltrate (without alveolar consolidation) on chest radiograph to cases with evidence of alveolar consolidation (without evidence of an other infiltrate). No significant differences were found for any virus after adjusting for site and age.

## DISCUSSION

In the PERCH study, the evidence for the utility of NP/OP viral load in distinguishing radiographically confirmed cases of severe or very severe pneumonia from controls was mixed. On the one hand, we found a higher mean viral load in NP/OP samples from severe and very severe pneumonia cases than from community controls without pneumonia for several respiratory viruses. Moreover, for many viruses, using a higher viral load threshold to define positivity that maximized the combination of sensitivity and specificity increased the odds ratio for case status over a simple binary (presence/absence) definition of positivity based on viral detection, which has high sensitivity but low specificity. On the other hand, there was substantial overlap in the distribution of NP/OP viral load densities among cases and controls, even for RSV, the virus most strongly associated with case status. No cutoffs clearly distinguished cases from controls.

Previous studies have also shown that the median or mean RSV concentration of NP/OP specimens among children is higher in cases of severe illness than among a healthy or mildly ill control population [7–10, 20, 21]. However, few of these studies compared the distribution of viral loads between severe cases and controls. Those that did compare these 2 groups showed an overlapping distribution similar to our study [8, 22]. Some studies of RSV viral load failed to show an association with severe lower respiratory tract infection [23, 24], but these studies included older children and adolescents in whom the pathogenic significance of detecting RSV in the NP/OP is less clear. One study of RSV viral load in infants showed no association with severe bronchiolitis [25] while another suggested that viral load only influences clinical severity for first RSV infections in young infants [10].

In some studies, influenza viral load was associated with severe disease [4, 26–29], while in others there was no association [8, 23, 27, 30–32]. A few studies have shown a higher viral load in severe cases for HMPV [24, 33, 34]. Higher viral loads of HBOV in nasopharyngeal aspirates were associated with greater severity of illness among Chinese children [35]. Rhinovirus viral load has been associated with more severe illness [22], but not in some studies [8, 21]. Again, the majority of these studies looked only at the central tendency of the viral load and did not demonstrate a clear dichotomy in the distribution of viral loads based on case status or severity category.

We did not find a higher viral load associated with greater severity among pneumonia cases for most viruses. Cases who died had a similar viral load as those who survived, and those with very severe pneumonia had similar viral loads to those children with severe pneumonia. This is in contrast with some other studies of viruses in which higher viral load was observed among RSV-infected children requiring mechanical ventilation [9], and severe acute respiratory syndrome coronavirus– and Middle East respiratory syndrome coronavirus–infected adults who died [36, 37].

We undertook this analysis, in part, to determine if viral loads of NP/OP specimens could be incorporated into the PERCH analysis to identify etiologies of severe/very severe pneumonia. Using a higher density threshold also did not have an appreciable effect on the population attributable fraction for most viruses, suggesting that using higher thresholds to assign viral etiology to cases would likely have little impact on the analysis of the etiologic distribution among the population of PERCH cases [38]. In the final PERCH analyses, we will be able to run sensitivity analyses to assess the impact of incorporating viral density thresholds on the assessment of etiology.

In contrast to the accompanying analyses of bacterial pneumonia, our conclusions about the interpretation of viral load and whether to include it in the main PERCH etiology analysis were limited by the lack of a gold standard to diagnose viral pneumonia [5, 16]. There were few PERCH cases who underwent lung aspirate procedures and even fewer who had a lung aspirate in which a virus was detected in their lungs. Among a population of pneumonia cases in which a virus was detected in the NP/OP, there was likely a mixture of those in whom the virus had a causal role in pneumonia and those in whom it did not. The inability to identify which children had pneumonia due to which virus hampered the study’s ability to determine if higher viral loads in the NP/OP were associated with pneumonia.

Besides the lack of a gold standard, other limitations might have affected our results. First, specimens were taken at one point in time on admission to the hospital. We observed that viral load varied with the time since illness onset, with higher viral load earlier in the course of symptomatic illness for some viruses. Taking sequential samples in which we could compare the peak viral load between cases of different clinical severity would have been optimal. Second, our design precluded us from assessing the role of viral load in the lung. Upper respiratory tract viral load might reflect the amount of replication in the local epithelial cells rather than the viral burden in the lung parenchyma. Evaluation of viral load of specimens from the lung, either through lung aspirates or bronchoalveolar lavage, would provide more direct evidence of the role of viral load in pneumonia severity.

The widespread use of sensitive PCR assays for testing NP/OP specimens has led to a higher reported prevalence of pneumonias attributed to respiratory viruses in both adults and children [2]. Due to the high prevalence of viral infections of the URT itself, however, it is difficult to conclude that detection of a virus in the URT of a pneumonia patient is equivalent to having pneumonia due to that virus. In the PERCH study, the viral loads in the NP/OP of pneumonia patients are unlikely to further clarify the role of that virus in causing pneumonia. However, the PERCH study design was not optimal to answer this question definitively. Further research, such as longitudinal studies and animal models, is needed to better elucidate the interpretation of viral load in the diagnosis and clinical management of viral pneumonia.

## Supplementary Data

Supplementary materials are available at *Clinical Infectious Diseases* online. Consisting of data provided by the author to benefit the reader, the posted materials are not copyedited and are the sole responsibility of the author, so questions or comments should be addressed to the author.

## Supplementary Material

Supplemental Tables Figures

## Notes

***Author contributions.*** D. R. F. led the analysis, interpreted results, and drafted initial manuscript. W. F. and Q. S. performed the analysis. W. F., Q. S., D. E. P., M. M. H., and D. R. M. provided significant guidance on the development of the manuscript. O. S. L., K. L. O., D. R. F., L. L. H., R. A. K., D. R. M., M. D. K., H. C. B., W. A. B., S. R. C. H., K. L. K., S. A. M., J. A. G. S., D. M. T. conceived and designed the study and supervised study conduct. W. F., D. E. P., Q. S., M. M. H., P. V. A., M. A., J. O. A., V. L. B., A. N. D., A. J. D., B. E. E., D. G., M. L., S. C. M., J. M., J. M., C. P., P. S., S. O. S., M. D. T., T. W., and K. Z. were involved in study conduct. S. L. Z. provided statistical expertise and led the integrated etiology analysis. All authors reviewed and approved the manuscript. D. R. F. had full access to all the data in the study and had final responsibility for the decision to submit for publication.

***Acknowledgments.*** We acknowledge the significant contributions of the PERCH Study Group and all PERCH investigators. We offer our gratitude to the members of the Pneumonia Methods Working Group, PERCH Expert Group, and PERCH Chest Radiograph Reading Panel for their time and lending expertise to assist the PERCH Study Group. See Supplementary Materials for a full list of names. We offer sincere thanks to the patients and families who participated in this study.

***Financial support.*** PERCH was supported by the Bill & Melinda Gates Foundation (grant number 48968 to the International Vaccine Access Center, Department of International Health, Johns Hopkins Bloomberg School of Public Health). J. A. G. S. was supported by a clinical fellowship from the Wellcome Trust of Great Britain (award number 098532).

***Supplement sponsorship.*** This article appears as part of the supplement “Pneumonia Etiology Research for Child Health (PERCH): Foundational Basis for the Primary Etiology Results,” sponsored by a grant from the Bill & Melinda Gates Foundation to the PERCH study of Johns Hopkins Bloomberg School of Public Health, Baltimore, Maryland.

***Potential conflicts of interest.*** M. D. K. has received funding for consultancies from Merck, Pfizer, and Novartis, and grant funding from Merck. L. L. H. has received grant funding from Pfizer and GlaxoSmithKline. K. L. K. has received grant funding from Merck Sharp & Dohme. S. A. M. has received honoraria for advisory board membership from the Bill & Melinda Gates Foundation, Pfizer, Medimmune, and Novartis; has received institutional grants from GSK, Novartis, Pfizer, Minervax, and the Bill & Melinda Gates Foundation; and has served on speaker’s bureaus for Sanofi Pasteur and GSK. K. L. O. has received grant funding from Pfizer and GlaxoSmithKline and has served on technical advisory boards for Merck, Sanofi Pasteur, PATH, Affinivax, and ClearPath. All other authors report no potential conflicts. All authors have submitted the ICMJE Form for Disclosure of Potential Conflicts of Interest. Conflicts that the editors consider relevant to the content of the manuscript have been disclosed.
