# Supplementary material for: Is Higher Viral Load in the Upper Respiratory Tract Associated With Severe Pneumonia? Findings From the PERCH Study
Source: Clin Infect Dis. 2017 May 29;64(Suppl 3):S337–46. doi: 10.1093/cid/cix148 (PMC5447843; doi:10.1093/cid/cix148)
Supplement: Supplemental Tables Figures [file cix148_suppl_Supplemental_Tables_Figures.docx]

Supplementary Table 1. Association between Nasopharyngeal/Oropharyngeal Cycle Threshold (Ct) Values and Duration of Illness^a^ among CXR+^b^ Cases

|  | **1-2 days since onset** | |  | **3-6 days since onset** | |  | **> 7 days since onset** | |  | **p-value^c^** | | |
| --- | --- | --- | --- | --- | --- | --- | --- | --- | --- | --- | --- | --- |
| **Virus** | **n^d^** | **Ct Value**  **Mean (95% CI)** |  | **n^d^** | **Ct Value**  **Mean (95% CI)** |  | **n^d^** | **Ct Value**  **Mean (95% CI)** |  | **1-2 vs.**  **3-6 days** | **3-6 vs.**  **>7 days** | **1-2 vs.**  **>7 days** |
| **Adenovirus** | 58 | 29.5 (28.2-30.8) |  | 77 | 26.8 (25.5-28.0) |  | 28 | 26.4 (24.7-28.2) |  | .008 | .96 | .02 |
| **Coronavirus 229** | 4 | 32.9 (30.1-35.8) |  | 11 | 29.8 (26.4-33.2) |  | 3 | 33.1 (26.6-39.5) |  | .15 | .26 | . |
| **Coronavirus 43** | 13 | 28.0 (24.7-31.3) |  | 15 | 25.4 (22.1-28.6) |  | 9 | 25.4 (20.7-30.0) |  | .11 | .91 | .91 |
| **Coronavirus 63** | 13 | 26.9 (23.6-30.1) |  | 14 | 27.1 (24.8-29.3) |  | 9 | 27.0 (23.1-30.9) |  | .59 | .26 | .12 |
| **Coronavirus HKU** | 10 | 30.3 (25.6-35.1) |  | 15 | 29.0 (25.6-32.4) |  | 12 | 28.6 (24.7-32.4) |  | .86 | .75 | .54 |
| **Influenza A** | 13 | 30.3 (28.5-32.1) |  | 31 | 28.0 (26.9-29.0) |  | 17 | 28.2 (26.3-30.2) |  | .13 | .51 | .07 |
| **Influenza B** | 4 | 26.9 (23.1-30.8) |  | 8 | 27.3 (24.4-30.1) |  | 6 | 28.6 (24.9-32.4) |  | .44 | . | .08 |
| **Influenza C** | 1 | 24.5 (.-.) |  | 6 | 29.5 (24.2-34.7) |  | 2 | 27.6 (23.5-31.6) |  | .91 | .56 | . |
| **HBOV** | 80 | 30.6 (29.2-32.0) |  | 112 | 30.0 (28.8-31.3) |  | 38 | 31.2 (29.6-32.8) |  | .54 | .32 | .67 |
| **HMPV A/B** | 51 | 28.1 (27.1-29.0) |  | 114 | 27.9 (27.2-28.7) |  | 19 | 29.5 (28.1-30.9) |  | .43 | .29 | .52 |
| **Parainfluenza 1** | 31 | 26.0 (24.1-27.9) |  | 50 | 25.4 (23.9-26.8) |  | 8 | 30.8 (27.8-33.8) |  | .20 | .02 | .11 |
| **Parainfluenza 2** | 5 | 32.6 (26.7-38.6) |  | 10 | 31.9 (28.0-35.7) |  | 8 | 37.5 (36.1-38.8) |  | .55 | .32 | .70 |
| **Parainfluenza 3** | 31 | 23.9 (22.2-25.6) |  | 52 | 24.7 (23.5-25.9) |  | 21 | 27.1 (24.5-29.6) |  | .49 | .04 | .21 |
| **Parainfluenza 4** | 15 | 32.1 (29.5-34.8) |  | 21 | 31.0 (29.1-32.8) |  | 8 | 32.9 (29.4-36.5) |  | .62 | .51 | .59 |
| **PV/EV** | 51 | 30.3 (29.3-31.3) |  | 60 | 29.9 (28.9-30.9) |  | 20 | 30.3 (28.6-32.0) |  | .49 | .65 | .78 |
| **Rhinovirus** | 150 | 31.2 (30.6-31.7) |  | 154 | 31.9 (31.3-32.5) |  | 61 | 32.5 (31.8-33.1) |  | .18 | .98 | .16 |
| **RSV** | 108 | 21.6 (20.9-22.3) |  | 290 | 22.1 (21.7-22.6) |  | 62 | 23.4 (22.3-24.6) |  | .51 | .04 | .04 |

^a^ Duration of illness determined by longest duration of symptoms among cough, shortness of breath or fever.

^b^ Chest x-ray positive (CXR+) defined as having radiographic evidence of pneumonia

^c^ P-values obtained comparing mean Ct values after adjusting for age and site. Missing p-values are a result of zero cells.

^d^ Number positive in the nasopharynx for that virus.

Supplementary Table 2. Mean Nasopharyngeal/Oropharyngeal PCR Cycle Threshold (Ct) Values among Chest X-ray (CXR) positive cases by HIV status, South Africa and Zambia Sites

| **Virus** | **HIV Negative^a^, CXR+^b^ Cases (N=622)** | | | **HIV Positive, CXR+^b^ Cases (N=142)** | | |  |
| --- | --- | --- | --- | --- | --- | --- | --- |
|  | **N** | **%** | **Ct Value Mean (95% CI)** | **N** | **%** | **Ct Value Mean (95% CI)** | **p-value (adjusted)^c^** |
| **Adenovirus** | 82 | 9.9 | 27.2 (26.1-28.3) | 22 | 15.6 | 27.0 (24.8-29.2) | .63 |
| **Coronavirus 229** | 8 | 1.0 | 30.1 (27.1-33.2) | 1 | 0.7 | 35.0 (.) | .37 |
| **Coronavirus 43** | 18 | 2.2 | 27.8 (25.0-30.6) | 5 | 3.5 | 24.4 (18.3-30.6) | **.04** |
| **Coronavirus 63** | 18 | 2.2 | 26.3 (23.6-29.0) | 3 | 2.1 | 31.0 (26.6-35.5) | .55 |
| **Coronavirus HKU** | 20 | 2.4 | 28.3 (25.3-31.3) | 3 | 2.1 | 27.4 (24.1-30.8) | .67 |
| **Influenza A** | 31 | 3.7 | 28.4 (27.2-29.6) | 1 | 0.7 | 22.4 (.) | .18 |
| **Influenza B** | 9 | 1.1 | 27.7 (25.1-30.4) | 0 | 0.0 | . | . |
| **Influenza C** | 5 | 0.6 | 24.0 (22.5-25.6) | 2 | 1.4 | 23.4 (19.6-27.2) | .56 |
| **HBOV** | 113 | 13.7 | 31.1 (29.9-32.3) | 23 | 16.3 | 30.0 (27.3-32.7) | .44 |
| **HMPV A/B** | 73 | 8.8 | 28.0 (27.1-28.9) | 5 | 3.5 | 31.8 (27.8-35.8) | **.02** |
| **Parainfluenza 1** | 20 | 2.4 | 28.9 (27.5-30.3) | 4 | 2.9 | 30.5 (27.3-33.7) | .54 |
| **Parainfluenza 2** | 3 | 0.4 | 33.9 (27.1-40.7) | 1 | 0.7 | 32.1 (.) | . |
| **Parainfluenza 3** | 47 | 5.7 | 24.7 (23.3-26.2) | 13 | 9.2 | 28.1 (25.3-30.8) | **.01** |
| **Parainfluenza 4** | 21 | 2.5 | 31.4 (29.3-33.5) | 4 | 2.8 | 35.2 (32.5-37.9) | .11 |
| **PV/EV** | 50 | 6.0 | 29.6 (28.8-30.4) | 5 | 3.5 | 32.3 (28.8-35.7) | .32 |
| **Rhinovirus** | 195 | 23.6 | 31.2 (30.8-31.6) | 29 | 20.6 | 32.1 (31.1-33.2) | .19 |
| **RSV** | 236 | 28.5 | 22.0 (21.4-22.5) | 18 | 12.8 | 27.0 (23.7-30.3) | **<.001** |

^a^ Includes 155 children without clear documentation of negative status but who are believed to be HIV-negative.

^b^ CXR+ defined as having radiographic evidence of pneumonia.

^c^ Comparing mean Ct value in HIV positive vs. HIV negative cases adjusting for age and site. Missing p-values are a result of zero cell

Supplementary Figure 1. Nasopharyngeal/Oropharyngeal Viral Density (log10 copies/mL) for Controls with and without Symptoms of Respiratory Tract Illness (RTI) among those with Positive Densities


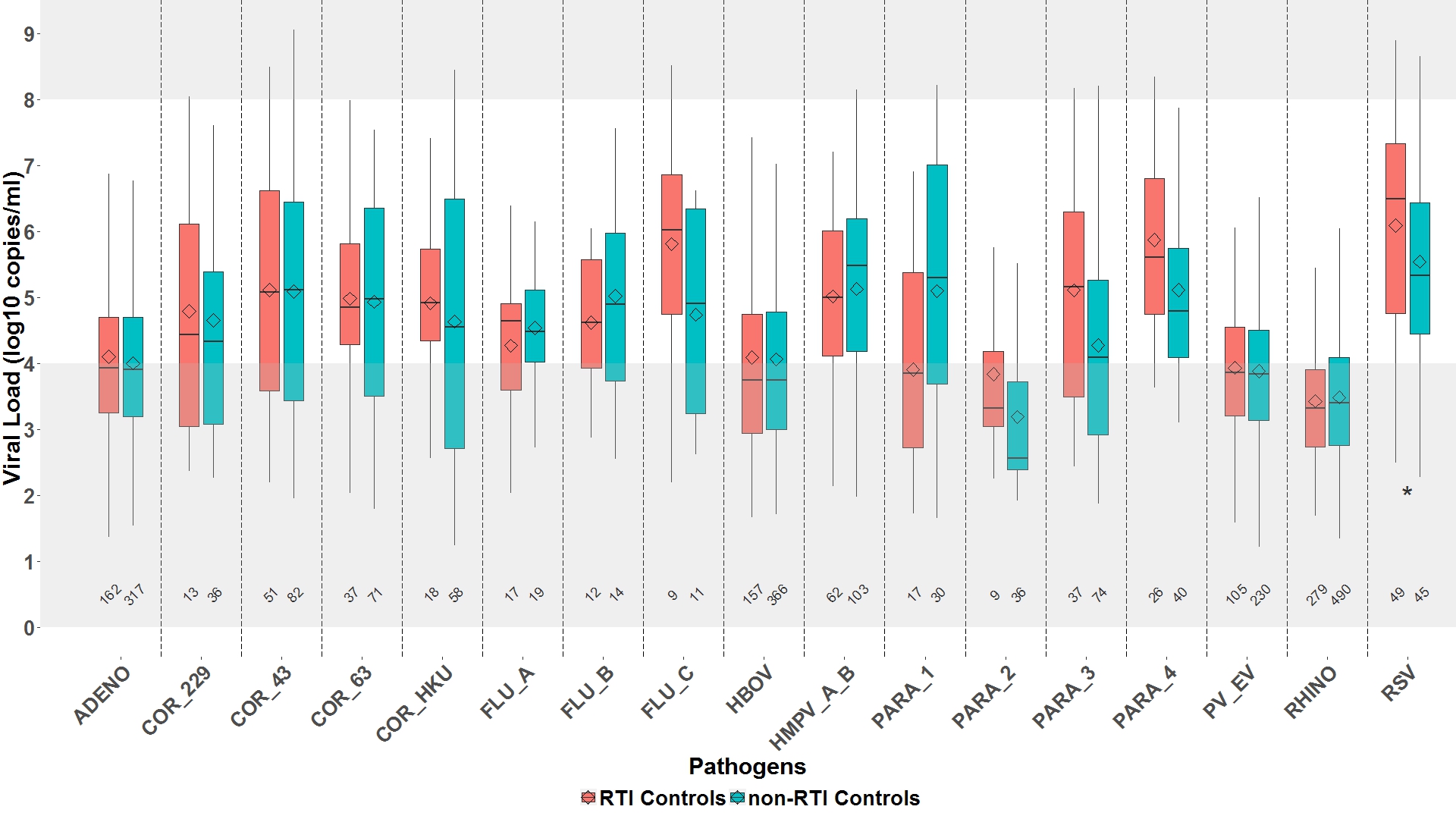
 Restricted to 5 of 7 PERCH sites (excluding Zambia and South Africa which had few RTI controls). Box and whiskers plot features include the following: central line in box is median, bottom line of box is first quartile (25%), top line of box is third quartile (75%), diamond is mean, top and bottom of whiskers represent 95% confidence intervals. Shaded areas indicate areas outside the linear range of the assay for calculation of viral load from Ct values, where there is a greater degree of uncertainty in viral density calculations. Numbers on x-axis indicate number of positive results for that virus.

*p-value comparing means between RTI and non-RTI controls <.05 after adjusting for age and site.

Supplementary Figure 2. Linear Regression of Nasopharyngeal/Oropharyngeal Viral Density (log10 copies/mL) on Age for CXR+ Cases and Controls among those with Positive Densities, by Age


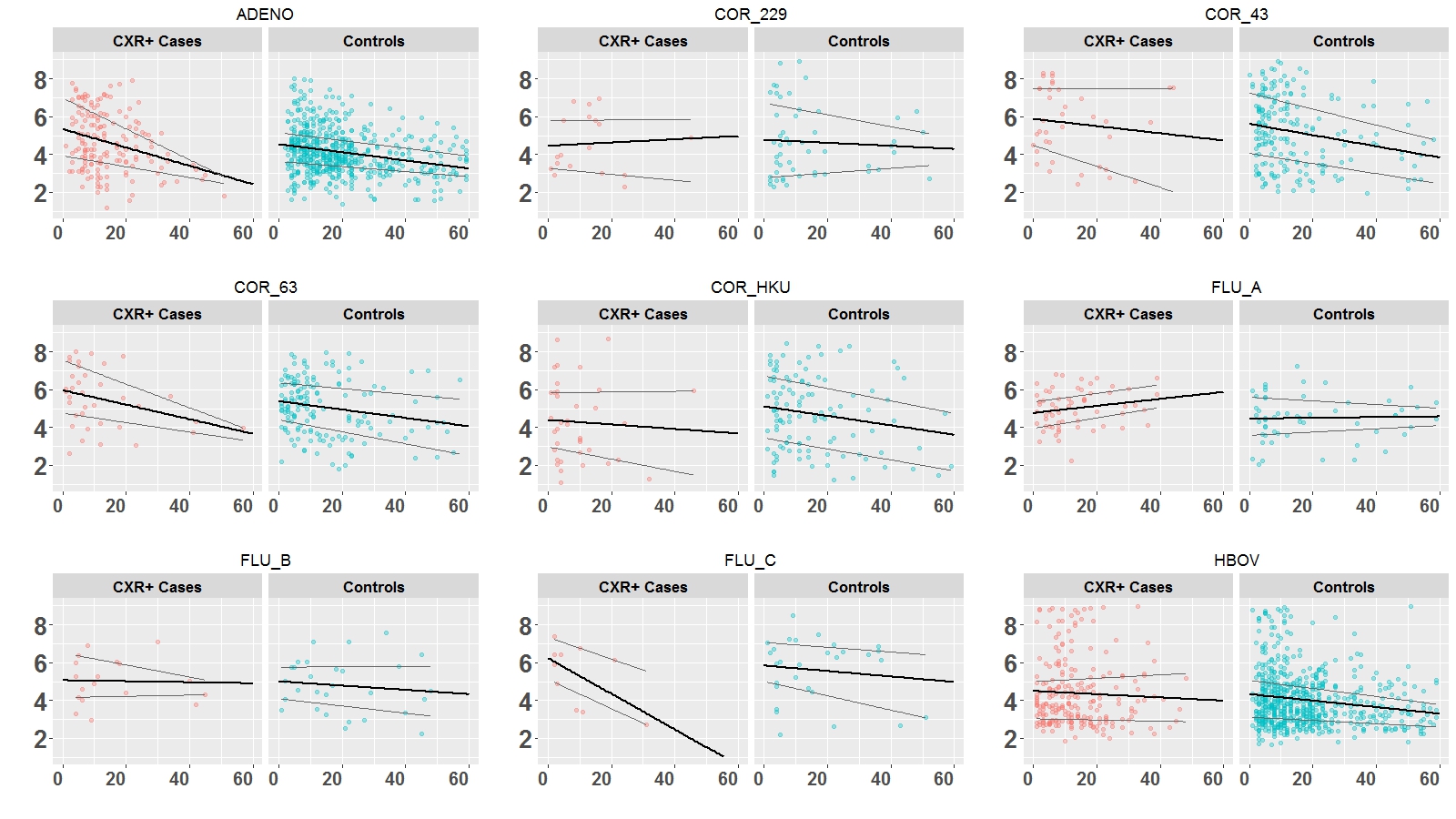


**Age (months)**

**Age (months)**

**Age (months)**

**Viral Density (log10 copies/ml)**

**Viral Density (log copies/ml)**

**Viral Density (log copies/ml)**

Supplementary Figure 2 (*continued*). Linear Regression of Nasopharyngeal/Oropharyngeal Viral Density (log10 copies/mL) on Age for CXR+ Cases and Controls among those with Positive Densities, by Age


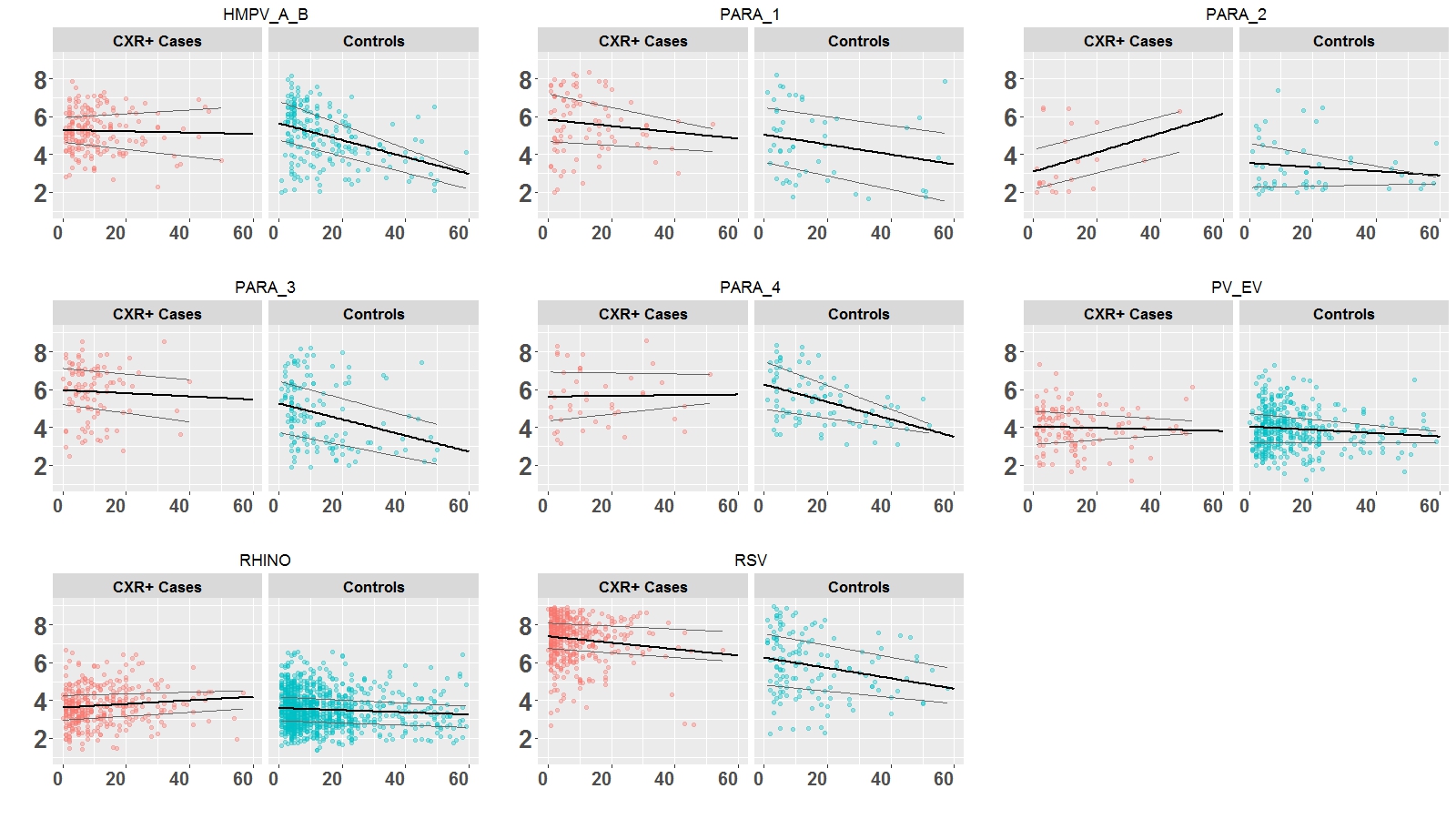


**Age (months)**

**Age (months)**

**Age (months)**

**Viral Density (log copies/ml)**

**Viral Density (log copies/ml)**

**Viral Density (log10 copies/ml)**

Chest x-ray positive (CXR+) defined as having radiographic evidence of pneumonia. Black line: simple linear regression of NP/OP viral density on age. Grey lines: fitted 25% and 75% quantiles of the viral density using quantile regression. For CXR + cases, a significant trend of decreasing viral load with increasing age was observed for adenovirus and RSV and a significant trend of increasing viral load with increasing age was observed for rhinovirus after adjusting for site. For controls, a significant trend of decreasing viral load with increasing age was observed for adenovirus, coronavirus 43, coronavirus HKU, influenza B, influenza C, parainfluenza viruses 3 and 4, parechovirus/enterovirus, rhinovirus, and RSV after adjusting for site.

Supplementary Figure 3. Nasopharyngeal/Oropharyngeal Viral Density (log10 copies/mL) among CXR+ Cases with Positive Densities, by Pneumonia Severity


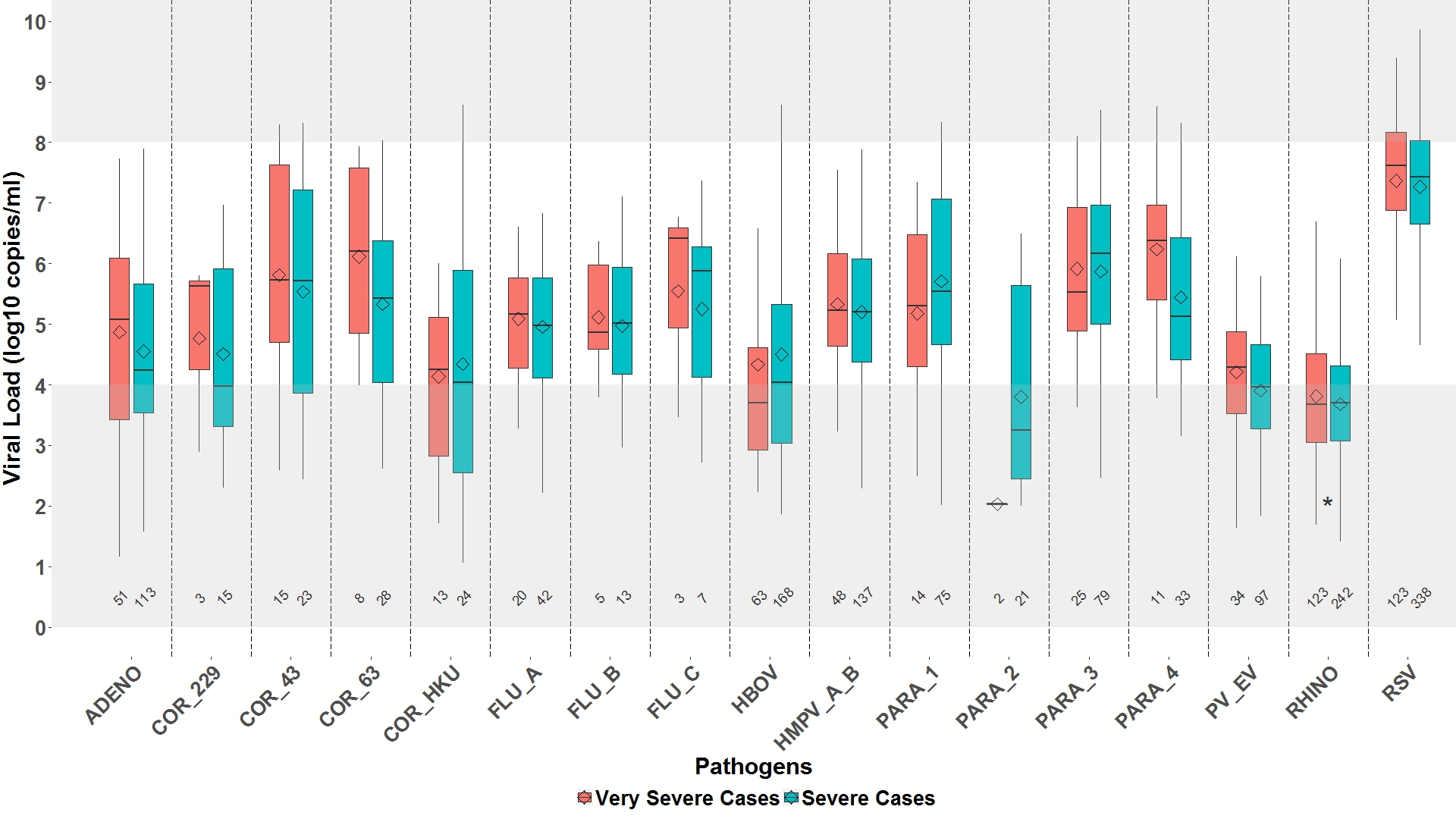
Chest x-ray positive (CXR+) defined as having radiographic evidence of pneumonia. Box and whiskers plot features include the following: central line in box is median, bottom line of box is first quartile (25%), top line of box is third quartile (75%), diamond is mean, top and bottom of whiskers represent 95% confidence intervals. Shaded areas indicate areas outside the linear range of the assay for calculation of viral load from Ct values, where there is a greater degree of uncertainty in viral density calculations. Numbers on x-axis indicate number of positive results for that virus.

*p-value comparing means between severe and very severe cases < .05 after adjusting for age and site.

Supplementary Figure 4. Nasopharyngeal/Oropharyngeal Viral Density (log10 copies/mL) for CXR+ Cases among those with Positive Densities, by Vital Status


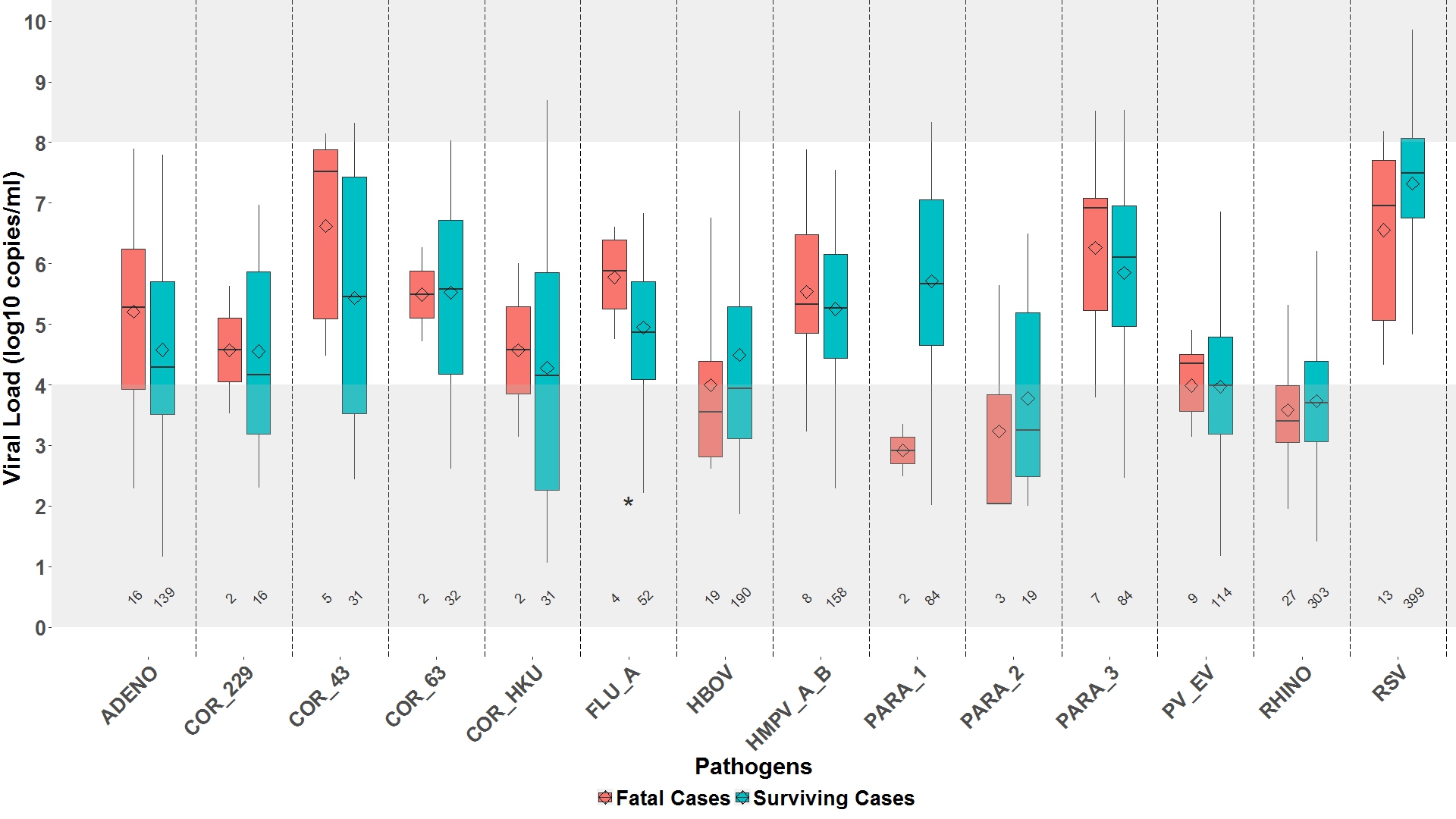


Chest x-ray positive (CXR+) defined as having radiographic evidence of pneumonia. Box and whiskers plot features include the following: central line in box is median, bottom line of box is first quartile (25%), top line of box is third quartile (75%), diamond is mean, top and bottom of whiskers represent 95% confidence intervals. Shaded areas indicate areas outside the linear range of the assay for calculation of viral load from Ct values, where there is a greater degree of uncertainty in viral density calculations. Numbers on x-axis indicate number of positive results for that virus. Influenza B, parainfluenza 1, and parainfluenza 4 excluded due to no positive results in at least one of the comparison categories.

*p-value comparing means between fatal and surviving cases <.05 after adjusting for age and site.

**Abbreviations for Supplementary Figures 1-4:**

ADENO, adenovirus; COR_229, human coronavirus 229; COR_43, human coronavirus 43; COR_63, human coronavirus 63; COR_HKU, human coronavirus HKU; FLU_A, influenza A; FLU_B, influenza B; FLU_C, influenza C; HBOV, human bocavirus; HMPV_A_B, human metapneumovirus A/B; PARA_1, parainfluenza virus 1; PARA_2, parainfluenza virus 2; PARA_3, parainfluenza virus 3; PARA_4, parainfluenza virus 4; PV_EV, parechovirus/enterovirus; RHINO, rhinovirus; RSV, respiratory syncytial virus.

**Acknowledgements:**

***PERCH Expert Group.*** William C. Blackwelder, Harry Campbell, John A. Crump, Adegoke Falade, Menno D. de Jong, Claudio Lanata, Kim Mulholland, Shamim Qazi, Cynthia G. Whitney.

***Pneumonia Methods Working Group.*** Robert E Black, Zulfiqar A Bhutta, Harry Campbell, Thomas Cherian, Derrick W Crook, Menno D de Jong, Scott F Dowell, Stephen M Graham, Keith P Klugman, Claudio F Lanata, Shabir A Madhi, Paul Martin, James P Nataro, Franco M Piazza, Shamim A Qazi, and Heather J Zar.

***PERCH Chest Radiograph Reading Panel***

**Readers:** Dr. Kamrun Nahar, Dr. Fariha Bushra Matin, Dr. Claire Oluwalana, Dr. Bernard Ebruke, Dr. Joyce Sande, Dr. Micah Silaba Ominde, Dr. Mahamadou Diallo, Dr. Breanna Barger-Kamate, Dr. Nasreen Mahomed, Dr. David P. Moore, Dr. Anchalee Kruatrachue, Dr. Piyarat Suntarattiwong, Dr. Musaku Mwenechanya, Dr. Rasa Izadnegahdar, **Arbitrators:** Dr. Vera Manduku, Dr. John DeCampo, Dr. Marg DeCampo, Dr. Fergus Gleeson.

***PERCH Contributors:***

**Bangladesh:** Kamrun Nahar, Arif Uddin Sikdir, Sharifa Yeasmin, Dilruba Ahmed, Muhammad Ziaur Rahman, Muhammad Yunus, Muhammad Al Fazl Khan, Muhammad Jubayer Chisti, Abu Sadat Muhammad Sayeem, Shahriar Bin Elahi, Mustafizur Rahman; **The Gambia:** Michel Dione, Emmanuel Olutunde, Peter Githua, Ogochukwu Ofordile, Rasheed Salaudeen, David Parker; **Kenya:** Shebe Mohamed, Siti Ndaa, Micah Silaba, Neema Muturi, Angela Karani, Sammy Nyongesa, Anne Bett, Daisy Mugo, Salim Mwarumba, Robert Musyimi, Andrew Brent, James Nokes, David Mulewa, Joyce Sande, John Odhiambo, Joshua Wambua, Nuru Kibirige, Caroline Mulunda, Hellen Mjalla, Norbert Katira, Karen Dama, Loice Masha, Christine Mutunga, Mwanajuma Ngama, Stephen Mangi, Riziki Anthony, Mwarua Yubu, Elijah Wakili, Benson Katana, Shoboi Mgunya, Emmanuel Mumba, Benedict Mver, George Kuria, Felix Githinji, Norbert Kihuha, Boniface Jibendi, Tahreni Bwanaali, Agustus Kea; **Mali:** Nana Kourouma, Aliou Toure, Mahamadou Diallo, Breana Barger-Kamate, Mariam Samake, Seydou Sissoko, Abdoul Aziz Maiga, Mariam Samake, Toumani Sidibe, Mariam Sylla, Aziz Diakite, Bassirou Diarra; **South Africa:** Azwidihwi Takalani, Andrea Hugo, Susan Nzenze, Ndulela Titi, Mmabatho Selela, Malebo Motiane, Minah Nkuna,Nonhlanhla Tsholetsane, Sibonsile Moya, Debra Katisi, Tondani Netshishivhe, Lerato Mapetla, Gudani Singo, Simphiwe Gasa, Cece Mgenge, Nozipho Mthunzi, Nombulelo Monedi, Tanja Adams, Shafeeka Mangera, Jeannette Wadula, Peter Tsaagane, Jenifer L. Vaughan, Sakina Loonat, Martin Hale, Sugeshnee Pather, Mariëtte Middel, Siobhan Trenor, Palesa Morailane, Ntombi Maya, Rene Sterley, Charné Combrinck, Given Malete, Lerato Qoza, Grizelda Liebenberg, Hendrik van Jaarsveld, Zunaid Kraft, Lisa-Marie Mollentze, Lourens Combrinck, Tsholofelo Mosome; **Thailand:** Sununta Henchaichon, Dr. Tussanee Amornintapichet, Dr. Somchai Chuananont, Toni Whistler, Juraiporn Ratanodom, Patranuch Sapchookul, Ornuma Sangwichian, Sirirat Makprasert, Manoon Hirunsalee, Possawat Jorakate, Anek Kaewpan, Duangkamol Siludjai, Apiwat Lapamnouysup, Dr. Wantana Paveenkittiporn, Waraporn Ubonphen, Dr. Peera Areerat, Ms.Yupapan Wannachaiwong, Ms Tewa Faipet, Ms Punnat Natnarakorn, Ms Ahchanan Sacharone, Mr.Winai Makmool, Ms. Kanlaya Sornwong, Ms. Promporn Sansuriwong, Ms. Ratchanida Potiya, Ms. Wasana Hongsawong, Ms.Wipa Matchaikhen, Ms. Thatsanawan Chaiyabil, Ms.Piyapai Wannarach, Ms Chamaiporn Wadeesirisak, Mr. Yuttapong Norapet, Mattana Bangkung, Mr. Barameht Piralam, Sathapana Naorat, Anchalee Jatapai, Prasong Srisaengchai, Dr. Leonard Peruski, Ms.Dawan Phaensoongnoen, Ms.Tussaaorn Klangprapan, Ms.Narawadee Dumrongdee, Ms.Atchara Srithongkham, Mr. Piyawut Noinont, Ms. Pornthip Kamlee, Ms.Siyapa Mongkornsuk; **Zambia:** Justin Mulindwa, Musaku Mwenechanya, John Mwaba, Magdalene Mwale, Julie Duncan, Kazungu Siazele, Muntanga Mapeni, Emily Hammond; **Canterbury Health Laboratory, Christchurch, New Zealand:** Rose Watt, Shalika Jayawardena.
